# Mutualistic Relationships between Microorganisms and Eusocial Wasps (Hymenoptera, Vespidae)

**DOI:** 10.3390/microorganisms11051340

**Published:** 2023-05-19

**Authors:** Stefano Turillazzi, Niccolò Meriggi, Duccio Cavalieri

**Affiliations:** 1 Department of Biology, University of Firenze, Via M. del Piano 6, 50019 Firenze, Italy; niccolo.meriggi@unifi.it (N.M.); duccio.cavalieri@unifi.it (D.C.); 2 Joint Laboratory LABREMMA, University of Firenze, Via M. del Piano 6, 50019 Firenze, Italy; 3 Insect Pharma Entomotherapy s.r.l., Via M. del Piano 6, 50019 Firenze, Italy

**Keywords:** social wasps, viruses, bacteria, fungi, yeasts, antimicrobial secretions, mutualistic symbioses

## Abstract

Eusocial wasps are represented in the Vespidae by the subfamilies Stenogastrinae, Vespinae and Polistinae. These wasps present colonies that are sometimes composed of thousands of individuals which live in nests built with paper materials. The high density of the adult and larval population, as well as the stable micro environment of the nests, make very favourable conditions for the flourishing of various types of microorganisms. These microorganisms, which may be pathogens, are beneficial and certainly contribute to model the sociality of these insects. The mutualistic relationships that we observe in some species, especially in Actinomycete bacteria and yeasts, could have important fallouts for the development of new medicines and for the use of these insects in agricultural environments.

## 1. Introduction

Eusociality, the condition in which a group of individuals presents communal cares of immature brood, generations overlap and, above all, some members of the group renounce exploiting their reproductive capacity to rear the offspring of other (often related) individuals, is not so represented in nature [1]. In insects, we can find it especially in Termites and Aculeate Hymenoptera (ants, bees and wasps). Considering the wasps (family Vespidae), it has been asserted, in light of phylogenetic studies (based on data from four nuclear genes fragments [2] on sequence data generated by 378 loci across 136 vespid species [3] and on mtgenome PCG12R datasets [4]), that eusociality originated twice: once in the subfamily Stenogastrinae and the other in the ancestor of the group composed of the subfamilies Polistinae and Vespinae.

The relationship between wasps and microorganisms of various types (viruses, bacteria and unicellular fungi) is present in solitary species, but, during evolution, it acquired a special and diverse significance in social species. The main characteristic of the latter is the formation of colonies that can have populations spanning from a few individuals to large superorganisms. Adults and immature broods live in nests that are mainly built with materials collected in the field and treated with a gluing secretion of the adults before being used for construction. The colony (represented by the nest, adults and immature brood) forms special environments where microorganisms can proliferate, which can present challenges to the lives of these insects, meaning that we can expect the presence of the various systems evolved by the hosts to limit or influence the pathogens and commensals in their nests [5,6]. Recently, the focus on the microbiome has led to us considering an individual as the product of the interaction of its genes and the genes of the microorganisms inhabiting its body. The hologenome theory [7,8], which regards all microorganisms and the host as the unique subject exposed to selective pressure, has been extensively described in insects. Among these, Aphids and *Buchnera* have improved our understanding of the evolutionary dynamics in host–microorganism interactions [9], microorganism symbiosis studies on *Nasonia* highlighted the role of the microbiota in the speciation process [10], while studies on termites were crucial for the synthesis and degradation of nutrients from plant polymers [11].

Social insects are a perfect example of the holobiont theory of evolution since they depend significantly on commensal yeasts, fungi and bacteria (Guerrero et al., 2013) [12]. With respect to other social insects, however, reports and experiments on the symbiotic relationships between social wasps and microorganisms are quite limited. Recently, Mayorga et al., 2021 [13], published an excellent review that is part of a book on South American social wasps, which focuses on the published contributions about the presence of microorganisms in the colonies of these insects. The review also presents synoptic tables which list the viruses, bacteria and fungi found mainly in Vespinae and Polistinae wasps. At present, more than 150 species of microorganisms have been reported to be present in the colonies of social wasps, with a vast majority of fungi (almost 70%) (Majorga et al., 2021 [13].

The purpose of this short review is to give an account of the principal examples of the mutualistic symbiosis between social wasps and microorganisms reported in the literature. First, however, we must mention at least one of the kinds of defence evolved by social wasps against pathogenic microorganisms.

## 2. Defence against Pathogens: Antimicrobial Secretions

The defence of social wasps against pathogens evolved in various ways both at the individual and social level [14]: the choice of where to build the nests, the organisation of nest architecture, the presence of hygienic behaviours and the production of antimicrobial substances. The last ones are particularly important as they can be effective for the individuals and the whole colony [15], and can be present in the secretions of special glands of the larvae and the adults, or produced by mutualistic microorganisms. In all the cases, the products can be of great interest for the development of antimicrobial agents [16]. All the species in which the presence of these substances was searched for had a colony defence based on active substances which can be secreted by the larvae ([17] for *Vespula (Vla)* sp. and [18] for *Polistes dominula*). The venom, however, was especially found to be the source of important compounds. The venom of social insects is a very complex secretion which contains components of various molecular weights; the medium weight components are mainly formed by short peptides, of a few residues spanning from 12 to 15, that are called mastoparans [19,20]. Mastoparans can cause many different effects on biological organisms and possess cytolytic and antimicrobial activity. Various types have been described in the venom of social wasps belonging to several species of Stenogastrinae [15], Vespinae (*Vespa tropica*—[21]; *V. magnifica*, *V. orientalis*, *V. nigrithorax*—[22]; *Vespula vulgaris*—[23]; *Dolichovespula saxonica*—[24]) and Polistinae (*Agelaia pallipes pallipes*—[25]; *Polybia paulista*—[26]; *Polybia dymorpha*—[27]; *Chartergellus communis*—[28]; *Sinoeca surinama*—[29]; *Polistes dominula* [30]; *P. major major* and *P. dorsalis dorsalis*—[31]; and *P. wattii*—[32]). Moreover, targets of the antimicrobial activity span from bacteria to fungi and even viruses. Hoggard et al. in 2011 [33] noted that the antimicrobial activity of social species, which build paper nests, tends to be the highest with respect to that of solitary species, with an increment also related to group size and social complexity.

## 3. The Eusocial Wasps

### 3.1. The Stenogastrinae: The Primitively Eusocial Wasps

**Figure 1:**
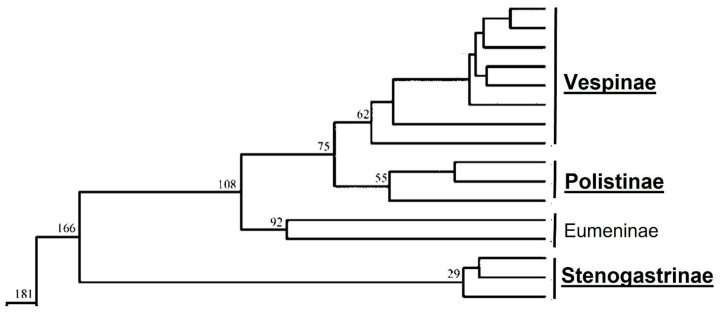
Simplified phylogenetic tree and evolutionary timescale of Vespidae inferred from mtgenome PCG12R Protein Coding Genes datasets. Eusociality evolved twice, once in the Stenogastrinae and once in Vespinae + Polistinae. Numbers represent millions of years. Four non-eusocial subfamilies (Gayellinae, Euparaginae, Masarinae, Zethinae) are not considered (from Huang et al., 2019 [4], redrawn).

Owing to Huang et al. [4], the Stenogastrinae are the sister group of all the other subfamilies of Vespidae, and their splitting from them dates to 166 Mys (Figure 1). The group presents small size colonies and a primitive eusociality, while differences between fertile and sterile individuals are only behavioural. The “hover wasps” (a name given for their hovering flight) include seven genera which live in forest environments and are limited to Eastern tropical Asia (from India to Indonesia and the Philippines) and the Papuan Region. Their geographical distribution, of course, can cause some problems for the development of research so that many species are almost certainly still unknown. Turillazzi [34] gave a general account of their morphology, biology and behaviour. Information on the presence of microorganisms in their small colonies is quite scanty and deals uniquely with the observation that the nests of some species (*Eustenogaster eximia* [35] and *Anischnogaster laticeps* [36]) present the hyphae of some fungi which cross the material of the nest walls, offering a reinforcement to the nest structure. At present, other information on possible mutualistic symbioses are lacking. Attempts to detect microorganisms on nest walls and in the “pap” (Dufour’s gland secretion), which serves to rear the larvae or protect the nest from small predators, did not ascertain the presence of microorganisms, but at the same time, failed to determine the antimicrobial action of some glands [37]. This was instead proven for the venom of species of at least three genera (*Liostenogaster*, *Eustenogaster* and *Parischnogaster)*, which the adults simply smeared on their bodies during cleaning movements [15]. Research on the microbiomes of adults and larvae is lacking at the present, but these wasps represent an interesting source of studies for evolutionary microbiologists owing to their small colonies, multi-variate nest architecture and primitive social behaviours.

### 3.2. The Polistinae and Vespinae: The More Evolute Eusocial Wasps

Owing to Huang et al. [4], the second independent origin of eusociality in Vespidae occurred approximately 75 Mys ago; Polistinae and then Vespinae evolved from a eusocial ancestor and achieved a more complete eusociality with the development of a system of caste determination based on the differentiation of queens and workers at the preimaginal stage. Polistinae is the group with the largest number of genera (25, according to Silveira et al. [38], divided into four tribes: Polistini, Mischocyttarini, Epiponini and Ropalidiini), while Vespinae comprise species of four genera (*Vespa*, *Vespula (Vla.)*, *Dolichovespula* and *Provespa*). In the case of living Polistinae, we can observe an increase in colony size from species with a limited number of individuals to quite large superorganisms, while the latter state is the rule for all the living species of Vespinae. Notwithstanding, these wasps have been studied far more than the Stenogastrinae, and only some genera received an attention pointed at characterising their relationships with microorganisms. We decide to mention the reported cases focusing on the kind of associated microorganisms: viruses, bacteria and fungi.

#### 3.2.1. Viruses

While viruses associated with honeybees are largely known, those that interest social wasps are, for the most part, unknown. In some solitary parasitoid wasps (Ichneumonids and Brachonids [39]) (for a review see Roossinck [40]), mutualistic important viruses (Polydnaviruses) prepare the host organism for the colonisation by the eggs of the parasitoid; however, the presence of viruses in the colonies of social wasps has hardly been reported, certainly because they have not yet been accurately looked for. Morel and Fouillaud [41] examined the meconia and guts of the larvae in nests of *Polistes olivaceus* (previously *P. hebraeus*) from the Reunion Island, finding inclusion bodies which released *Baculovirus* and *Reovirus* particles. In a second paper [42], they described Cypoviruses (CPV) and Nucleopolyendroviruses (NPV) similar to those found in the Lepidoptera, which are preys of the wasps. The authors concluded that the nests of Vespidae could be used for the study of populations of insect viruses in biotopes. *Polistes* wasps are also considered possible carriers for the virus of grape disease [43]. Dalmon et al. [44] searched for viruses in the Asian invasive hornet *Vespa velutina* and found 19 species, the most abundant of which was the DWV (Deformed Wing Virus) of honeybees, and concluded that the largest part of the species belonged to the preys of the hornets. At present, these are the only studied relationships between viruses and social wasps. It is quite possible that a wider and more complex series of interactions will be discovered in the future, owing to advanced microbiome studies made possible by more and more powerful instruments and techniques. In any case, no mutualistic relationship has been described so far.

#### 3.2.2. Bacteria

The mutualistic relationships between bacteria and social wasps have been studied, but the studies remain far less numerous than those performed on other social insects, including, for example, studies conducted on the relationships which occur in colonies of leaf cutter ants (*Atta* sp. and *Acromyrmex* sp.). Here, complex interactions between insects, bacteria and fungi have been described and deeply studied [45,46,47].

Research on the microbiota of some social wasps heightened our understanding of the possible relationships between bacteria and these insects [48,49,50]. The group of Cini et al. [50] analysed adults of various ages and castes, life stages and nest parts of the invasive social wasp *Vespa velutina nigrithorax*, with targeted metagenomics aimed at the characterisation of bacteria and fungi. They found Bacilli, Gammaproteobacteria, Actinobacteria and Alphaproteobacteria to be the most representative classes of bacteria. More dated research is again that of Morel and Fouillard [41], who searched for microorganisms in the meconia and guts of the larvae in the nests of the only social wasps present in the Reunion Island (*Polistes olivaceus = P. haebreus*). They recognised 12 species of *Bacillus* and various species of Enterobacteriacaee, Pseudomonadacee and Gram-positive Cocci. 

The first to propose a mutualistic relationship between bacteria and social wasps was probably Jacob Ishay and co-workers [51] in a paper on *Vespa orientalis*. They noticed that the silk of the pupating larvae, which is produced by their labial glands, is accompanied by bacteria that protect the pupae and, thereafter, facilitate the emergence of the adults by practicing holes in the silk texture of the cocoons. They identified strains of two species of *Staphylococcus* (*S. arlette* and *S. cohnii*). The authors noted that the bacteria were transmitted from one host generation to the subsequent generation through trophallactic interactions (which are the passages of food from adults to larvae and salivary secretions from larvae to adults). This finding is noteworthy as *Staphylococcus* is considered a pathogenic microorganism for man and other animals.

In an unpublished thesis of the Biological Sciences courses of the University of Florence in 2006, Tempestini [52] reported the presence of eight genera of Actinomycetes (*Kokuria, Rothia, Micrococcus*, *Agrococcus*, *Microbacterium*, *Corynebacterium*, *Streptomyces)* in the nests of the European *Polistes dominula*. The production of active antimicrobial substances from symbiotic Actinomycetes extracted from nests of the same species (invasive in United States and in other extra-European countries) was then demonstrated by Madden et al. [53], who tested 30 isolates belonging to the genera *Streptomyces*, *Micromonospora* and *Actinoplanes* against pathogenic *Pseudomonas ruginosa*, *Escherichia coli*, *Staphylococcus aureus, Serratia marcescens* and *Bacillus subtilis.* Owing to Mayorga et al. [13], this finding adds a further explanation to the reasons why this invasive species seems to be advantaged with respect to some indigenous American species [54]. Since the research of Madden et al. in 2013 [52], however, no study of this kind has been performed on other species of the genus *Polistes* with the exception, in 2018, of the doctoral thesis of T.R. Mhlongwe [55], who reported a species of *Bacillus* isolated from the nest of the invasive *P. dominula* in the Western Cape Region of South Africa, which inhibits the growth of the entomopathogenic fungus *Beauveria bassiana.*

Various studies, however, confirmed the importance that the symbiosis between Actinomyceta and insects has for the possible development of new medicines. Chevrette et al. [56] asserted that the strains of *Streptomyces* extracted from insects have more antibacterial activity than that of the strains found in soil or on plants. Baranova et al. [57] gave an account of the techniques to extract Actinomycetes from insects and on the antimicrobial activity of the chemical compounds produced by these microorganisms.

For social wasps, Matarrita-Carranza [58] examined the presence and supposed the antimicrobial activity of 197 Actinobacteria isolates (most of the genus *Streptomyces*) from the nests of various social insects, including species of the social Epiponine wasps (a tribe of Polistinae) *Agelaia cayannensis*, *Metapolybia docilis*, *Polybia plebeja*, *Polibya occidentalis bohemani* and other not determined Vespidae. Chavarria-Pizarro [59] found 36 strains of antimicrobic Actinomycetes extracted from the breeding cells (larvae and larval meconia) of the nests of species of five genera (*Parachartergus*, *Chartergellus*, *Matapolybia*, *Polybia*, *Protopolybia*) of Epiponine wasps. The group of Matarrita-Carranza [60] demonstrated the production of antimicrobial compounds against a pathogenic fungus (*Hirsutella citriformis*) and the human pathogens *Staphylococcus aureus* and *Candida albicans* by a *Streptomyces* sp. M54 associated with the social wasp *Polybia plebeja*.

More recently, Gutierrez et al. [61], with the aim to search for new natural products with a wide range of competing activity with insect pathogens, examined the Actinomycetes found on the cuticle and in the salivary glands of adults of two species of Epiponine social wasps found in Costa Rica, belonging to the genera *Methapolybia* and *Protopolybia*. The bacteria were identified as species of the genera *Streptomyces* (from *Protopolybia*) and *Saccharopolyspora* (from *Methapolybia*). The authors furnished the genome sequences of six bacteria, but did not ascertain their biological activity.

Mutualistic relationships between bacteria and social wasps remain a wider and promising field of study. For what we know at present, species of Actinomycetes, like other social insects, represent the most important microorganisms for the production of substances with defensive activity against pathogens invading their colonies. This, however, must still be ascertained for the species of Polistinae of the tribe Ropalidiini (genera *Parapolybia*, *Belonogaster*, *Ropalidia e Polybioides*), where the larval meconia are eliminated by the adults after larval pupation.

#### 3.2.3. Fungi

The fungi of social wasps probably received the most attention from researchers. Pathogenic species, of course, were the most described, with the aim to detect possible limiting agents against insect pests [62,63]. This was true, for example, for species of the genus *Metharizium* and *Beauveria*, but in some cases, interesting examples of mutualism were discovered and described.

In 1965, Durrell [64] was the first to describe the presence of fungi in the paper of a nest of a species of *Vespula* fallen in the surroundings of his house. The author limited his research to the recognition of at least six species of fungi: *Aerobodisium pollutans*, *Phoma* sp., *Fusarium roseum*, *Mucor varians*, *Alternaria tenuis* and *Stemphylium ilicis*, concluding that wasps had brought them to the nest together with the construction material. He observed that, in any case, the fungal hyphae contributed to reinforcing the nest walls. As we have already seen, this is also present in at least two species of Stenogastrinae [35,36]. Fouillard and Morel [65] later confirmed the presence of 52 fungal species of 31 genera (including moulds and yeasts) on the bodies and meconia of the larvae in the nests of *Polistes olivaceus (=P. haebreus)*. *Aspergillus* and *Penicillus* were the most abundant together, with potentially entomopathogenic and phytopathogenic species. Similarly, Jayaprakash and Ebenezer [66], while describing the mycobiota of the Indian paper wasp *Ropalidia marginata*, found species of *Aspergillus* and *Penicillus*. Madden et al. [67] even described a new fungal species, *Mucor nidicola*, isolated from the nest of *Polistes dominula*, but they did not find, nor search for, any mutualistic relationships between these fungi and the insects. 

Davis et al. [68] were the first to discover and experimentally test that the fungus *Aerobasidium pollulas*, which is usually found in decomposing fruits, emits volatile substances (mainly 2-methyl1-butanol and others) which attract social yellowjackets wasps (*Vespula germanica* and *V. pensylvanica*) to food. The fungus, on its own, is vectored in the environment by the wasps. This is an example of mutualistic symbiosis, which has been confirmed and analysed for various yeasts.

**Figure 2:**
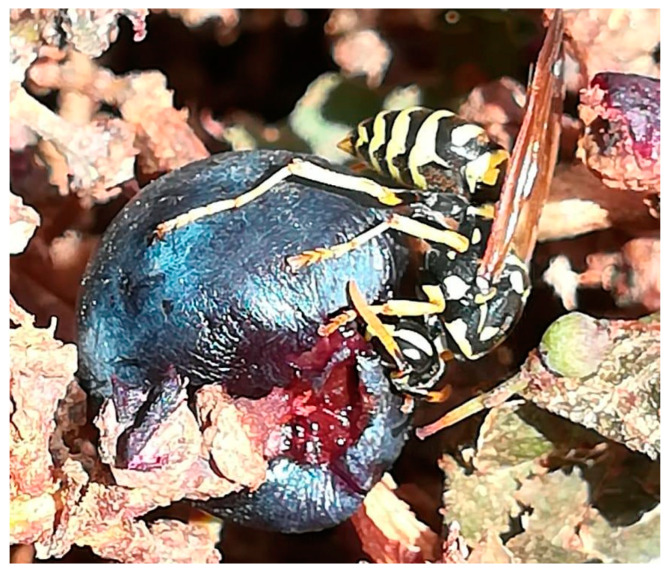
Social wasp *Polistes dominula* foraging on ripe grapes to acquire sugars. The photo was taken at the end of August in Castagneto Carducci (LI), Tuscany, Italy.

Yeasts and social wasps maintain special mutualistic relationships of ecological and economical importance. The situation is nicely summarised by the title of Blackwell [69]: “Made for each other: ascomycete yeasts and insects”. In 2012, a group belonging to the University of Firenze, composed of entomologists and microbiologists, discovered that the annual cycle of *Saccharomyces cerevisiae*, which is at the basis of the fermentation of various products used in human alimentation, occurs in the gut of social wasps [70]. More detailed research of the same group ascertained that in the paper wasp *Polistes dominula* and in the hornet *Vespa crabro*, *S. cerevisiae* can undergo sexual reproduction and form hybrids that are not present in the field with other *Saccharomyces* [71] (Figure 2). Dapporto et al. [72] confirmed the wide range of phenotypic variability of *S. cerevisiae* in a particular geographic area, and evaluated the differential production of volatile metabolites, observing that these could influence its ability to attract insects.

The mutualistic relationships between yeast and insects can develop on various lines. On part of the fungi, their cells, too heavy to be carried by the wind, have the opportunity to be vectored by the insects into the environment. Insects, on the other hand, receive a special benefit from carrying microorganisms. The largest part of the information related to the communication between *S. cerevisiae* and the insect immune system was obtained using *Drosophila melanogaster*, a well-known insect model used in the study of toll-like receptors [73,74]. Immune trials performed on the Lepidoptera *Galleria mellonella* also provided crucial information on the response of the immune system against yeast cell wall structures, highlighting the increased response of this insect against *Candida albicans* infection after pre-exposure to beta-glucans [75]. While social wasps are usually not considered in immune—yeast interaction studies, a paper by Meriggi et al. [76] has demonstrated that specific strains of *S. cerevisiae* (already proven to induce trained immunity in mammals [77]) were able to enhance the immune system of the paper wasp *P. dominula*, increasing the bacterial clearance against the pathogen *E. coli*. This latest work has laid the foundations for a more accurate consideration of the evolution of the interactions between insects and yeasts (Figure 2).

Different species of yeast, rather than *S. cerevisiae*, can be hosted in the gut of social wasps in other geographical areas; Jimenez et al. [78], working in British Columbia (Canada), found yeasts of the genera *Lachancea*, *Hanseniaspora* and *Metschnikowia,* but not *Saccharomyces*, in all the stages of *Vespula germanica*, *Vla. consombrina*, *Vla. alascensis*, *Vla. pensylvanica* and *Dolichovespula maculata*. This does not mean that social wasps are unique carriers of yeast, but due to their biological and behavioural characteristics, they represent important carrying agents in the environment. This has been further stressed by Valentini et al. [79] who discovered that wasps collected in areas near forests present a higher number of yeast cells and a wider biodiversity than insects caught in more open areas. This is probably related to the fact that yeasts (including *S. cerevisiae*) can also be found in the bark of trees or in other natural substrata [70]. In any case, the study of this kind of symbiosis promises, for the future, very interesting results for a wider comprehension of the ecological systems and for important economic fallouts [80,81]. In a not-yet-published research (Di Paola et al. submitted), the females and males of *Polistes dominula* have been used to convey in a vineyard environment specific yeast strains in order to change the wine aromatic pattern. This confirms the potential of wasps for applicative interventions in agronomic contexts.

## 4. Conclusions

Table 1 summarises the mutualistic relationships observed and experimentally confirmed, at present, between microorganisms and social wasps. We can observe that the role of microorganisms in the various associations is principally defensive, with the production of antimicrobial substances, while the insects provide a house and propagation means to the organisms. Future research will probably discover other interesting characteristics of this symbiosis.

In conclusion, these insects, given the different stages of sociality they reached in the course of evolution, the characteristics of their nests which favour the presence of microorganisms, the production of antimicrobial compounds, the easy handling of the colonies of some species and other important ecosystem services they furnish [82], are crucial for the study of the interactions between different levels of biological entities (holobionts), the discovery of new medicines and the convey of useful microorganisms in the environment.

## Figures and Tables

**Table 1:** Characteristics of mutual symbiosis between social wasps and microorganisms. Only the experimental confirmed researches are reported.

Mutual Symbioses between Microorganisms and Social Wasps					
Contribute of Microorganisms			Contribute of Wasps		
Type of Service		Ref.	Type of Service		Ref.
Nest walls reinforcement	Fungal sp. Hyphae in nests of some Stenogastrinae and *Vespula* sp.	[35]	Micro-environment formation and stabilisation	Social wasp colonies constitute perfect environments for microorganisms	[5]
		[36]			[6]
		[64]			
Production of defensive substances against pathogens	Actinomycetes in nests of *Polistes dominula* and various Epiponini *Bacillus* sp. against *Beauveria* in *P. dominula*	[53]	Horizontal and vertical transmission of microorganisms to colony mates and immature brood	*P. dominula* and *Vespa crabro* on *Saccharomyces cerevisiae* *Vespa orientalis* on *Staphylococcus*	[70]
		[59]			[71]
		[60]			[51]
		[55]			
Production of attractants to food	*Aerobasidium pollulas* attracts *Vespulae* to decomposing fruits	[68]	Gut environment induces variability of microorganisms through sexual reproduction	*Polistes dominula* and *Vespa crabro* on *Saccharomyces cerevisiae*	[71]
Stimulation of immune system of the hosts	*Saccharomyces* on *Polistes dominula*	[76]	Carriers of microorganisms in the environment	*Vespula germanica* and *V. pensylvanica* are carriers of the fungus *A.pollulans. P. dominula* and *V.crabro* are carriers of *Saccharomyces*	[68]
					[70]
					[71]
					[72]
Defence of the pupae and facilitation of the emergence of the adults	*Staphylococcus* sp. in nests of *Vespa orientalis*	[51]			

## Data Availability

Data sharing is not applicable to this article.

## References

[1] Wilson E.O. (2019). Genesis: The Deep Origin of Societies.

[2] Hines H.M., Hunt J.H., O’Connor T.K., Gillespie J.J., Cameron S.A. (2007). Multigene phylogeny reveals eusociality evolved twice in vespid wasps. Proc. Natl. Acad. Sci. USA.

[3] Piekarski P.K., Carpenter J.M., Lemmon A.R., Moriarty Lemmon E., Sharanowski B.J. (2018). Phylogenomic evidence overturns current conceptions of social evolution in wasps (Vespidae). Mol. Biol. Evol..

[4] Huang P., Carpenter J.M., Chen B., Li T.J. (2019). The first divergence time estimation of the subfamily Stenogastrinae (Hymenoptera: Vespidae) based on mitochondrial phylogenomics. Int. J. Biol. Macromol..

[5] Boomsma J.J., Schmid-Hempel P. (2005). Pressure across the Major Groups of Social Insects. Insect Evolutionary Ecology, Proceedings of the Royal Entomological Society’s 22nd Symposium, Reading, UK, 2005.

[6] Hughes D.P., Pierce N.E., Boomsma J.J. (2008). Social insect symbionts: Evolution in homeostatic fortresses. Trends Ecol. Evol..

[7] Zilber-Rosenberg I., Rosenberg E. (2008). Role of microorganisms in the evolution ndof animals and plants: The hologenome theory of evolution. FEMS Microbiol. Rev..

[8] Rosenberg E. (2021). Evolution of holobionts: The hologenome concept. Microbiomes: Current Knowledge and Unanswered Questions.

[9] Shigenobu S., Yorimoto S. (2022). Aphid hologenomics: Current status and future challenges. Curr. Opin. Insect Sci..

[10] Zhu Z., Liu Y., Hu H., Wang G.H. (2022). Nasonia–microbiome associations: A model for evolutionary hologenomics research. Trends Parasitol..

[11] Berlanga M., Guerrero R. (2016). The holobiont concept: The case of xylophagous termites and cockroaches. Symbiosis.

[12] Guerrero R., Margulis L., Berlanga M. (2013). Symbiogenesis: The holobiont as a unit of evolution. Int. Microbiol..

[13] Mayorga-Ch D., Rodríguez-C C., Ortíz-Reyes A., Romero-Tabarez M., Sarmiento C.E., Prezoto F., Nascimento F.S., Barbosa B.C., Somavilla A. (2021). Interactions of Social Wasps in Microorganisms. Neotropical Social Wasps: Basic and Applied Aspects.

[14] Cremer S., Armitage S.A., Schmid-Hempel P. (2007). Social immunity. Curr. Biol..

[15] Baracchi D., Mazza G., Turillazzi S. (2012). From individual to collective immunity: The role of the venom as antimicrobial agent in the Stenogastrinae wasp societies. J. Insect Physiol..

[16] Erturk Ö., Bagdatli E. (2019). A comprehensive study on nest materials of *Vespa crabro* and *Polistes dominula*: Chemical properties and biological characterization with antioxidant and antimicrobial activity. Biologia.

[17] Gambino P. (1993). Antibiotic activity of larval saliva of Vespula wasps. J. Invertebr. Pathol..

[18] Turillazzi S., Perito B., Pazzagli L., Pantera B., Gorfer S., Tancredi M. (2004). Antibacterial activity of larval saliva of the European paper wasp *Polistes dominulus* (Hymenoptera, Vespidae). Insectes Sociaux.

[19] Hirai Y., Yasuhara T., Yoshida H., Nakajima T., Fujino M., Kitada C. (1979). A New Mast Cell Degranulating Peptide “Mastoparan” in the Venom of *Vespula lewisii*. Chem. Pharm. Bull..

[20] Choi M.B., Lee Y.H. (2020). The structure and antimicrobial potential of wasp and hornet (Vespidae) mastoparans: A review. Entomol. Res..

[21] Yang X., Wang Y., Lee W.H., Zhang Y. (2013). Antimicrobial peptides from the venom gland of the social wasp *Vespa tropica*. Toxicon.

[22] Kim J., Kim M., Lee M., Lee Y.J., Kim H.R., Nam J.O., Choi M.B., Hahn D. (2020). Antibacterial potential of Nidus vespae built by invasive alien hornet, *Vespa velutina nigrithorax*, against food-borne pathogenic bacteria. Entomol. Res..

[23] Kim Y., Son M., Noh E.-Y., Kim S., Kim C., Yeo J.-H., Park C., Lee K.W., Bang W.Y. (2016). MP-V1 from the Venom of Social Wasp Vespula vulgaris Is a de Novo Type of Mastoparan that Displays Superior Antimicrobial Activities. Molecules.

[24] Ertürk Ö., Başkan C., Koleren Z. (2018). Antimicrobial Activities of Nest Materials *Dolichovespula saxonica* (Fabricius, 1793) (Hymenoptera: Vespidae) in Turkey. Arıcılık Araştırma Derg..

[25] Wang K., Dang W., Xie J., Zhu R., Sun M., Jia F., Zhao Y., An X., Qiu S., Li X. (2015). Antimicrobial peptide protonectin (*Agelaya pallipes pallipes*) disturbs the membrane integrity and induces ROS production in yeast cells. Biochim. Biophys. Acta (BBA) Biomembr..

[26] Ribeiro S.P., Mendes M.A., Dos Santos L.D., de Souza B.M., Marques M.R., de Azevedo W.F., Palma M.S. (2004). Structural and functional characterization of N-terminally blocked peptides isolated from the venom of the social wasp *Polybia paulista*. Peptides.

[27] Das Neves R.C., Trentini M.M., de Castro e Silva J., Simon K.S., Bocca A.L., Silva L.P., Mortari M.R., Kipnis A., Junqueira-Kipnis A.P. (2016). Antimycobacterial activity of a new peptide polydim-I isolated from neotropical social wasp *Polybia dimorpha*. PLoS ONE.

[28] Lopes K.S., Campos G.A.A., Camargo L.C., de Souza A.C.B., Ibituruna B.V., Magalhães A.C.M., da Rocha L.F., Garcia A.B., Rodrigues M.C., Ribeiro D.M. (2017). Characterization of two peptides isolated from the venom of social wasp *Chartergellus communis* (Hymenoptera: Vespidae): Influence of multiple alanine residues and C-terminal amidation on biological effects. Peptides.

[29] Freire D.O., da Cunha N.B., Leite M.L., Kostopoulos A.G., da Silva S.N., de Souza A.C., Nolasco D.O., Franco O.L., Mortari M.R., Dias S.C. (2020). Wasp venom peptide, synoeca-MP, from Synoeca surinama shows antimicrobial activity against human and animal pathogenic microorganisms. Pept. Sci..

[30] Turillazzi S., Mastrobuoni G., Dani F.R., Moneti G., Pieraccini G., la Marca G., Nolasco D.O., Franco O.L., Mortari M.R., Dias S.C. (2006). Dominulin A and B: Two new antibacterial peptides identified on the cuticle and in the venom of the social paper wasp *Polistes dominulus* using MALDI-TOF, MALDI-TOF/TOF, and ESI-ion trap. J. Am. Soc. Mass Spectrom..

[31] Čeřovský V., Slaninová J., Fučík V., Hulačová H., Borovičková L., Ježek R., Bednárová L. (2008). New potent antimicrobial peptides from the venom of Polistinae wasps and their analogs. Peptides.

[32] Al-Shammery K., Hozzein W.N. (2022). Antibacterial activities of two potential peptides extracted from *Polistes wattii* Cameron, 1900 (Vespidae: Polistinae) wasp venom collected at Eastern Province, Saudi Arabia. PLoS ONE.

[33] Hoggard S.J., Wilson P.D., Beattie A.J., Stow A.J. (2011). Social complexity and nesting habits are factors in the evolution of antimicrobial defences in wasps. PLoS ONE.

[34] Turillazzi S. (2013). The Biology of Hover Wasps.

[35] Krombein K.V. (1991). Biosystematic Studies of Ceylonese Wasps, XIX: Natural History Notes in Several Families (Hymenoptera: Eumenidae, Vespidae, Pompilidae and Crabronidae).

[36] Hansell M.H., Turillazzi S. (1995). Nest structure and building material of three species of *Anischnogaster* (Vespidae Stenogastrinae) from Papua New Guinea. Trop. Zool..

[37] Turillazzi S. (1985). Function and characteristics of the abdominal substance secreted by wasps of the genus *Parischnogaster* (Hymenoptera Stenogastrinae). Monit. Zool. Ital. J. Zool..

[38] Silveira O.T., Andena S.R., Somavilla A., Carpenter J.M. (2021). Phylogeny and classification of the Neotropical social wasps. Neotropical Social Wasps: Basic and Applied Aspects.

[39] Kaltenpoth M., Engl T. (2014). Defensive microbial symbionts in Hymenoptera. Funct. Ecol..

[40] Roossinck M.J. (2011). The good viruses: Viral mutualistic symbioses. Nat. Rev. Microbiol..

[41] Morel G., Fouillaud M. (1992). Presence of microorganisms and viral inclusion bodies in the nests of the paper wasp Polistes hebraeus Fabricius (Hymenoptera, Vespidae). J. Invertebr. Pathol..

[42] Fouillaud M., Morel G. (1994). Characterization of cytoplasmic and nuclear polyhedrosis viruses recovered from the nest of *Polistes hebraeus* F. (Hymenoptera; Vespidae). J. Invertebr. Pathol..

[43] Madden A.A., Boyden S.D., Soriano J.A.N., Corey T.B., Leff J.W., Fierer N., Starks P.T. (2017). The emerging contribution of social wasps to grape rot disease ecology. PeerJ.

[44] Dalmon A., Gayral P., Decante D., Klopp C., Bigot D., Thomasson M., Herniou E.A., Alaux C., Le Conte Y. (2019). Viruses in the invasive hornet *Vespa velutina*. Viruses.

[45] Currie C.R. (2001). A community of ants, fungi, and bacteria: A multilateral approach to studying symbiosis. Annu. Rev. Microbiol..

[46] Little A.E., Currie C.R. (2007). Symbiotic complexity: Discovery of a fifth symbiont in the attine ant–microbe symbiosis. Biol. Lett..

[47] Moreau C.S. (2020). Symbioses among ants and microbes. Curr. Opin. Insect Sci..

[48] Kim E., Seo J., Yang S.H., Kim I.S., Koo Y. (2018). Intestine bacterial microbiota of Asian hornet (*Vespa velutina nigrithorax*) and honeybee. Korean J. Environ. Agric..

[49] Fang C., Achal V. (2020). Physico-Chemical Aspects and Complete Bacterial Community Composition Analysis of Wasp Nests. Sustainability.

[50] Cini A., Meriggi N., Bacci G., Cappa F., Vitali F., Cavalieri D., Cervo R. (2020). Gut microbial composition in different castes and developmental stages of the invasive hornet *Vespa velutina nigrithorax*. Sci. Total Environ..

[51] Ishay J.S., Riabinin K., Pertsis V. (2003). Symbiotic bacteria in hornet pupal silk. Naturwissenschaften.

[52] Tempestini A. (2006). Caratterizzazione di Batteri Associati a Larve di Polistes Dominulus.

[53] Madden A.A., Grassetti A., Soriano J.A.N., Starks P.T. (2013). Actinomycetes with antimicrobial activity isolated from paper wasp (Hymenoptera: Vespidae: Polistinae) nests. Environ. Entomol..

[54] Cervo R., Zacchi F., Turillazzi S. (2000). Polistes dominulus (Hymenoptera, Vespidae) invading North America: Some hypotheses for its rapid spread. Insectes Sociaux.

[55] Mhlongwe T.R. (2018). The Search for a Biological Control Agent to Control Invasive Polistes Dominula Wasps in the Western Cape Region, South Africa. Ph.D. Thesis.

[56] Chevrette M.G., Carlson C.M., Ortega H.E., Thomas C., Ananiev G.E., Barns K.J., Book A.J., Cagnazzo J., Carlos C., Flanigan W. (2019). The antimicrobial potential of Streptomyces from insect microbiomes. Nat. Commun..

[57] Baranova A.A., Zakalyukina Y.V., Ovcharenko A.A., Korshun V.A., Tyurin A.P. (2022). Antibiotics from Insect-Associated Actinobacteria. Biology.

[58] Matarrita-Carranza B., Moreira-Soto R.D., Murillo-Cruz C., Mora M., Currie C.R., Pinto-Tomas A.A. (2017). Evidence for widespread associations between neotropical hymenopteran insects and Actinobacteria. Front. Microbiol..

[59] Chavarría-Pizarro L. (2019). Los insectos y la biotecnología: Avispas sociales como fuente de nuevos compuestos antibióticos. Rev. Tecnol. Marcha.

[60] Matarrita-Carranza B., Murillo-Cruz C., Avendaño R., Ríos M.I., Chavarría M., Gómez-Calvo M.L., Tamayo-Castillo G., Araya J.J., Pinto-Tomás A.A. (2021). *Streptomyces* sp. M54: An actinobacteria associated with a neotropical social wasp with high potential for antibiotic production. Antonie Van Leeuwenhoek.

[61] Gutiérrez-Araya M., Núñez-Montero K., Pizarro-Cerdá J., Chavarría-Pizarro L. (2022). Draft Genome Sequences of *Saccharopolyspora* sp. Strains and *Streptomyces* sp. Strains, Isolated from Social Wasps (Vespidae; Polistinae: Epiponini). Microbiol. Resour. Announc..

[62] Glare T.R., Harris R.J., Donovan B.J. (1996). Aspergillus flavus as a pathogen of wasps, *Vespula* spp., in New Zealand. N. Z. J. Zool..

[63] Harris R.J., Harcourt S.J., Glare T.R., Rose E.A.F., Nelson T.J. (2000). Susceptibility of *Vespula vulgaris* (Hymenoptera: Vespidae) to generalist entomopathogenic fungi and their potential for wasp control. J. Invertebr. Pathol..

[64] Durrell L.W. (1965). Fungi in nests of paper wasps. Am. Midl. Nat..

[65] Fouillaud M., Morel G. (1995). Fungi associated with nests of the paper wasp *Polistes hebraeus* (Hymenoptera: Vespidae) on La Reunion Island. Environ. Entomol..

[66] Jayaprakash A., Ebenezer P. (2010). A new report on mycobiota associated with *Ropalidia marginata* paper nests. Indian J. Sci. Technol..

[67] Madden A.A., Stchigel A.M., Guarro J., Sutton D., Starks P.T. (2012). *Mucor nidicola* sp. nov., a fungal species isolated from an invasive paper wasp nest. Int. J. Syst. Evol. Microbiol..

[68] Davis T.S., Boundy-Mills K., Landolt P.J. (2012). Volatile emissions from an epiphytic fungus are semiochemicals for eusocial wasps. Microb. Ecol..

[69] Blackwell M. (2017). Made for each other: Ascomycete yeasts and insects. Microbiol. Spectr..

[70] Stefanini I., Dapporto L., Legras J.L., Calabretta A., Di Paola M., De Filippo C., Viola R., Capretti P., Polsinelli M., Turillazzi S. (2012). Role of social wasps in *Saccharomyces cerevisiae* ecology and evolution. Proc. Natl. Acad. Sci. USA.

[71] Stefanini I., Dapporto L., Berná L., Polsinelli M., Turillazzi S., Cavalieri D. (2016). Social wasps are a *Saccharomyces* mating nest. Proc. Natl. Acad. Sci. USA.

[72] Dapporto L., Stefanini I., Rivero D., Polsinelli M., Capretti P., De Marchi P., Viola R., Turillazzi S., Cavalieri D. (2016). Social wasp intestines host the local phenotypic variability of *Saccharomyces cerevisiae* strains. Yeast.

[73] Alarco A.M., Marcil A., Chen J., Suter B., Thomas D., Whiteway M. (2004). Immune-deficient *Drosophila melanogaster*: A model for the innate immune response to human fungal pathogens. J. Immunol..

[74] Lionakis M.S. (2011). *Drosophila* and *Galleria* insect model hosts: New tools for the study of fungal virulence, pharmacology and immunology. Virulence.

[75] Bergin D., Murphy L., Keenan J., Clynes M., Kavanagh K. (2006). Pre-exposure to yeast protects larvae of *Galleria mellonella* from a subsequent lethal infection by *Candida albicans* and is mediated by the increased expression of antimicrobial peptides. Microbes Infect..

[76] Meriggi N., Di Paola M., Vitali F., Rivero D., Cappa F., Turillazzi F., Gori A., Dapporto L., Beani L., Turillazzi S. (2019). *Saccharomyces cerevisiae* induces immune enhancing and shapes gut microbiota in social wasps. Front. Microbiol..

[77] Rizzetto L., Ifrim D.C., Moretti S., Tocci N., Cheng S.C., Quintin J., Renga G., Oikonomou V., De Filippo C., Weil T. (2016). Fungal chitin induces trained immunity in human monocytes during cross-talk of the host with *Saccharomyces cerevisiae*. J. Biol. Chem..

[78] Jimenez S.I., Carroll C., Babcock T., Derstine N., Hadwin A., Moore M., Gries G. (2017). Yeasts harbored by vespine wasps in the Pacific Northwest. Environ. Entomol..

[79] Valentini B., Barbero F., Casacci L.P., Luganini A., Stefanini I. (2022). Forests influence yeast populations vectored by insects into vineyards. Front. Microbiol..

[80] Madden A.A., Epps M.J., Fukami T., Irwin R.E., Sheppard J., Sorger D.M., Dunn R.R. (2018). The ecology of insect–yeast relationships and its relevance to human industry. Proc. R. Soc. B Biol. Sci..

[81] Meriggi N., Di Paola M., Cavalieri D., Stefanini I. (2020). *Saccharomyces cerevisiae*–insects association: Impacts, biogeography, and extent. Front. Microbiol..

[82] Brock R.E., Cini A., Sumner S. (2021). Ecosystem services provided by aculeate wasps. Biol. Rev..

